# Serum β-secretase 1 (sBACE1) activity in subjective cognitive decline: an exploratory study

**DOI:** 10.1007/s11357-025-01523-x

**Published:** 2025-01-20

**Authors:** Carlo Cervellati, Alessandro Trentini, Valentina Rosta, Angelina Passaro, Gloria Brombo, Carlo Renzini, Gerhard Multhaup, Giovanni Zuliani

**Affiliations:** 1 Department of Translational Medicine and for Romagna, Università of Ferrara, Via Luigi Borsari 46, 44121 Ferrara, Italy; 2 Department of Environmental and Prevention Sciences, University of Ferrara, Via Luigi Borsari 46, 44121 Ferrara, Italy; 3 University Center for Studies On Gender Medicine, University of Ferrara, Via Luigi Borsari 46, 44121 Ferrara, Italy; 4 Associazione Sammarinese Di Geriatria E Gerontologia (ASGG), Piazza M. Tini N. 12, Dogana, San Marino, Republic of San Marino; 5 Integrated Program in Neuroscience, McGill University, Montreal, QC H3G 0B1 Canada; 6 Department of Pharmacology and Therapeutics, McGill University, Montreal, QC H3G 1Y6 Canada

**Keywords:** BACE1, Alzheimer’s disease, MCI, Subjective cognitive impairment

## Abstract

β-Secretase-1 (BACE1) plays a key role in the regulation of cerebral amyloid-β homeostasis, being involved in amyloidogenic and, as recently found, amyloidolytic pathways. Growing evidence indicates that increased serum BACE1 (sBACE1) activity might represent an early biomarker for Alzheimer’s disease. Here, we tested the hypothesis that an increase in sBACE1 activity may already occur in individuals with subjective cognitive decline (SCD). We found that sBACE1 activity was significantly higher in individuals with SCD (*n* 118) compared to cognitively normal subjects (controls, *n* 137) (*p* < 0.001). Moreover, compared with SCD, sBACE1 activity was even higher in patients affected by amnestic (*n* 179) or non-amnestic mild cognitive impairment (MCI) (*n* 99) (*p* < 0.001 and *p* 0.02, respectively). In all cases, the respective increase in sBACE1 activity was significant after adjustment for possible confounders including age, sex, and comorbidities. We also found a significant sexual dimorphism, with women affected by either type of MCI, but not by SCD, having higher levels of serum BACE1 activity compared to men. These results provide evidence supporting the potential use of sBACE1 activity as tool for blood-based screening of cognitively healthy individuals at clinical risk of MCI and dementia.

**Supplementary Information:**

The online version contains supplementary material available at 10.1007/s11357-025-01523-x.

## Introduction

Subjective cognitive decline (SCD) is a term used to describe a self-reported cognitive decline in individuals without objective evidence of cognitive impairment on standardized tests or clinical evaluation [1]. It refers to the experience of cognitive changes that are noticeable to the single individual, but do not meet the criteria for mild cognitive impairment (MCI) or dementia [1]. SCD has gained attention as an area of research and clinical interest due to its potential significance in identifying very early cognitive changes and predicting the risk of future cognitive decline. As reported by Wang et al. in their meta-analysis, SCD doubled the risk of developing cognitive disorders [2]; in particular, SCD conferred a 2.29-fold excess risk for cognitive impairment, and a 2.16-fold excess risk for dementia [2].

The development of novel pharmacological therapies for AD requires the recognition of cognitive decline in its early stages. Even considering the current lack of treatments able to reverse the initial pathological changes, it may be possible to prevent or delay the development of dementia in a segments of the population at risk of AD by modifying the exposure to common risk factors [3]. In light of these considerations, identifying biomarkers that may be altered in subjects with SCD and may predict the development of MCI or dementia is of paramount importance. There is a wealth of evidence suggesting that CSF and neuroimaging biomarkers may be already altered in SCD [4–6]; Aβ42 and tau, two well-established biomarkers in AD continuum, have been shown to be significantly different in SCD compared to controls [4] and are able to discriminate individuals with SCD who will decline over time from those who will not [5].

β-Secretase-1 (BACE1) plays a critical role in Aβ42 homeostasis [7–9]. The importance of BACE1 in AD is highlighted by various studies showing elevated activity and concentration in brain regions affected by Aβ deposition, as well as in the cerebrospinal fluid (CSF) of individuals with AD and MCI [10–12].

These findings prompted us, as well as other researchers, to investigate whether central nervous system (CNS) alterations of BACE1 may be reflected in the periphery. At present, the validated biomarkers for AD are CSF and neuroimaging-based measures, although their use is not widespread due to high costs, lack of extensive validation, and perceived invasiveness [13]. Blood-based biomarkers represent a well-recognized ideal alternative to the current approach, mostly as pre-screening tools to select subjects for clinical trials [13–15]. The data collected so far clearly suggest that serum (and plasma) activity of BACE1 may be a reliable candidate biomarker for the early diagnosis of AD. Shen et al. were among the first to report that plasma BACE1 activity was significantly higher in patients with AD and MCI, compared to controls (total sample, *n* 174) [10]. This study also validated BACE1 activity as a candidate biomarker by demonstrating its correlation with CSF Aβ42 and CSF tau levels, and its ability to predict the conversion of MCI to AD. Subsequently, we confirmed and extended these findings in two studies on AD and MCI (total sample, *n* 266 and *n* 463, respectively) [16, 17]. More recently, we found that the diagnostic accuracy for serum BACE1 (sBACE1) activity increased from 76 to 99%, when the diagnosis of MCI (due to AD) and AD was supported by core CSF biomarkers [18].

The capacity of peripheral BACE1 to early reflect the brain changes of AD pathology was previously shown in a study conducted on SCD individuals [19]; the authors showed that in these individuals there is a strong correlation between plasma BACE1 and cerebral burden of Aβ, as assessed by ultrasensitive neuroimaging techniques.

Considering all the above evidence, we hypothesize that sBACE1 could serve as an early biomarker in individuals at high risk of AD, even in the absence of objective cognitive impairment. To address this hypothesis, we assessed sBACE1 activity in a large sample of SCD, healthy controls, and both amnestic and non-amnestic MCI (aMCI and naMCI, respectively).

## Methods

### Participants

Five hundred thirty-three older individuals were included in the present study. The study sample included the following:

- One hundred thirty-seven subjects were classified as SCD. They had complaint of persistent cognitive decline, normal performance in detailed neuropsychological battery of tests (including Mini Mental Sate Examination (MMSE)), and a clinical dementia rating (CDR) score of 0.
- Two hundred seventy-eight MCI patients. MCI was defined as the presence of a documented deficit in memory and/or other cognitive domain, without (single domain) or with (multiple domain) impairment in other cognitive domains, in an individual who did not meet the clinical criteria for dementia [20]. One hundred seventy-nine patients had amnestic MCI (aMCI) and ninety-nine were non-amnestic MCI (naMCI).
- One hundred eighteen cognitively normal subjects, free of subjective cognitive decline and/or functional impairment (controls).

MCI and SCD outpatients were referred to the Memory Clinic of the Department of Internal Medicine, S. Anna University Hospital, Ferrara (Italy); cognitively normal individuals were referred to the Prevention Center of the University of Ferrara.

Exclusion criteria were as follows: age < 65 years; any unstable or severe medical condition (e.g., severe hepatic or renal disease, unstable cardiovascular disease, cancer); dementia; major psychiatric disorders such as uncontrolled depression and schizophrenia; use of non-steroidal anti-inflammatory drugs (NSAIDs), antibiotics, or steroids. Neuropsychological tests were carried out as previously described [16]. Personal data and medical history (e.g., hypertension, coronary heart disease (CHD), diabetes) were collected by trained personnel. Clinical chemistry analyses (serum B-12 vitamin and folate, liver, kidney and thyroid function tests, blood cell count) were routinely performed. The study was approved by the Local Ethic Committees (cod number: 170579 for MCI and SCD, and 140,288 for Controls). This study conforms to The Code of Ethics of the World Medical Association (Declaration of Helsinki, 1975) and was conducted according to guidelines for Good Clinical Practice (European Medicines Agency). Patients were informed and a written consent was obtained.

### BACE1 assessment

Peripheral blood samples were collected by venipuncture into Vacutainer® tubes without anticoagulant after overnight fasting. After 30 min of incubation at room temperature, the blood samples were centrifuged at 4650 g for 20 min, after which the resulting sera were collected and stored in single-use aliquots at − 80 °C until further analysis.

#### BACE1 substrate synthesis

The substrate was designed based on the method by Grüninger-Leitch et al., incorporating a chemical modification at the C-terminus [21]. The BACE substrate, SEVNLDAEFR, was labeled at the C-terminus with the fluorescent tag Lucifer Yellow (LY) and at the N-terminus with the quenching group Dabsyl, forming a fluorescence resonance energy transfer (FRET) donor–acceptor pair. When the peptide cleavage occurs, the distance necessary for the FRET to be effective (within 10 nm) is no longer maintained and the Dabsyl group is no longer able to quench the fluorescence of Lucifer Yellow: therefore, fluorescence is observed proportional to the enzyme concentration (Supplementary Fig. 1). Following the synthetic approach described by Grüninger-Leitch et al. (2002), the Dabsyl moiety was attached via a lysine side chain, while LY, a light-sensitive molecule, was added to the C-terminus in the final step of synthesis. A convergent synthesis strategy was employed, in which the peptide sequence H–K(Dabsyl)SEVNLDAEFRC-NH2 and the maleimido-functionalized LY were synthesized and purified separately. The subsequent Thiol–Michael addition reaction between the C-terminal cysteine’s sulfhydryl group and the maleimide group of LY yielded the final product, i.e., H–K(Dabsyl)SEVNLDAEFRC(MAL-LY)-NH2, with high purity and quantitative yield. Detailed synthesis information is provided in the original study [16].

#### BACE1 assay

The assay utilizes the fluorescence resonance energy transfer (FRET) technique, where an increase in fluorescence is detected following peptide substrate cleavage by BACE [22]. The substrate H–K(Dabsyl)SEVNLDAEFRC(MAL-LY)-NH2 possesses a fluorescent donor and a quenching acceptor, which remain in proximity when the substrate is intact, preventing fluorescence emission [16]. Upon cleavage by BACE1, the peptide fragments separate, causing the fluorescent donor to be released from quenching [16, 22]. As a result, the fluorescence intensity increases in direct correlation with BACE1 enzymatic activity.

Once prepared, the substrate was first dissolved in dimethyl sulfoxide at a stock concentration of 392 μM and stored in aliquots at − 20 °C for up to 3 months (no significant change in the basal signal was detected within this time). The substrate was directly diluted from the stock to a final concentration of 30 μM in 50 mM acetate buffer at pH 4.5, and protected from direct light until use. One hundred microliters of diluted substrate was dispensed in triplicate into the wells of a black, flat-bottom microplate. After a pre-incubation of 10 min at 37℃ in the dark, the reaction was started by adding 5 µL of undiluted serum, and fluorescence measurements were taken every 30 s for 20 min, using 430 nm as the excitation wavelength and 520 nm for emission, in a Tecan Infinite M200 (Tecan Group, Switzerland) microplate reader. The kinetics were used to calculate reaction rates (Relative Fluorescence Units per minute), which were then converted to enzyme units (U). To achieve this, the assay was performed as detailed above, substituting the wild-type enzyme (beta-secretase human, Sigma-Aldrich, Cat. No. S4195) in the range of 0.25–2 U in place of the serum. The standard was prepared from the stock solution (the starting concentration varies depending on the lot number) to obtain an intermediate concentration of 10 U/μl, in 20 mM HEPES buffer, 124 mM NaCl, pH 7.4. Then, five solutions at different concentrations (0.4 U/μl, 0.3 U/μl, 0.2 U/μl, 0.1 U/μl, 0.05 U/μl) were prepared in the same buffer and used for the assay by following the previous method. The prepared enzyme dilutions were prepared shortly before use in the assay and kept on ice until use. A control without enzyme was used to correct for background fluorescence. The enzymatic units were determined by interpolation with the standard curve by making a graph with background corrected RFU/min on the ordinate and concentration on the abscissa. The relation was linear and the resulting equation was used for deriving calculation of enzyme activity measured in serum.

These are the main analytical parameters of the assay [16]: intra-assay percentage coefficient of variation (%CV) = 6.5% (min–max, 2.6–10.9%); inter-assay %CV = 11.4% (min–max, 9.9–13%); intermediate precision = 10.5% (min–max, 6.3–13.5%). The intra-assay %CV was determined by assaying three serum samples with high, medium and low levels of BACE1 eight times (eight replicates) within the same run on two different occasions within the same day (in the morning and in the afternoon) by the same operator and using the same batches of substrate and buffer. For the determination of the inter-assay %CV, the same samples were assayed over a 3-week period by using the same batches of substrate and buffer and performed by the same operator. For the calculation of the intermediate precision, the samples were assayed by different operators over a 9-month period and by using different batches of substrate and buffer each time.

### Statistical analysis

Appropriate descriptive statistics including mean, standard deviation, median, and range were computed in presenting the overall data.

Continuous variables were tested for normality using both the Kolmogorov–Smirnov and Shapiro–Wilk tests.

Variables that met normality assumption were presented as mean ± standard deviation (see Table 1). Comparisons between the four study groups (controls, SCD, aMCI, and naMCI) were conducted using one-way analysis of variance (ANOVA). Post hoc comparisons, to detect between-group differences, were performed using the Bonferroni correction to adjust for multiple testing.

**Table 1:** Main characteristics of the sample

	Controls (*n* = 137)	SCD (*n* = 118)	naMCI (*n* = 99)	aMCI (*n* = 179)
Characteristics				
Age (years)	72 ± 4	76 ± 6^a^	77 ± 4^a^	78 ± 5^a^
Female gender	67 (49)	65 (55)	62 (62)^a^	97 (54)
Formal education (years)*	11 (8–13)	8 (5–13)^a^	5 (5–8)^a^	5 (5–7)^a,b^
MMSE score (/30)	27 (25–28)	28 (26–29)	25 (23–27)^a,b^	25 (23–26)^a,b^
Comorbidities				
Hypertension, *n* (%)	48 (35)	83 (70)^a^	70 (71)^a^	116 (65)^a^
Diabetes, *n* (%)	8 (6)	19 (16)^a^	14 (14)^a^	30 (17)^a^
CVDs, *n* (%)	5 (4)	9 (8)	15 (15)^a,b^	30 (17)^a,b^

Normally distributed variables are expressed as mean ± SD, while non-normally distributed variables are indicated as median (interquartile range). Categorical variables are expressed as number and percentage within groups. Statistical analysis: ANOVA for normal variables; Kruskal–Wallis for non-normal variables; chi-square for categorical variables. Pairwise comparison: ^a^*p* < 0.05 vs. controls; ^b^ < 0.05 vs. SCD; *SCD* subjective cognitive impairment, *MMSE* Standardized Mini-Mental State Examination, *CVD* cardiovascular diseases

For continuous variables that did not meet the normality assumption, data were expressed as median and interquartile range (IQR). Differences between the groups were assessed using the Kruskal–Wallis test. Pairwise comparisons were subsequently conducted using the Mann–Whitney *U* test, with Bonferroni correction applied to account for multiple comparisons.

Categorical variables were expressed as percentages. Differences in frequencies between groups were evaluated using the chi-square (*χ*^2^) test.

Associations between variables of interest were analyzed using Pearson’s correlation for normally distributed variables and Spearman’s rank correlation for non-normally distributed variables.

Since the distribution of sBACE1 activity was found to be normal across all four groups, as well as within the subsets of women and men, ANOVA was used to detect significant differences. To determine whether the observed differences were independent of potential confounders (age, hypertension, cardiovascular diseases, diabetes, and sex), analysis of covariance (ANCOVA) was performed. The ANCOVA is a statistical technique that combines the features of ANOVA and linear regression. It is usually employed to compare the means of three or more groups while controlling for the effects of one or more continuous covariates (named confounders or confounding variables). By adjusting for these covariates, ANCOVA helps to isolate the effect of the independent variable (the different group a subject belongs to) on the dependent variable (the level of sBACE1), providing a clearer understanding of the true relationship between them.

A two-tailed *p*-value of less than 0.05 was considered statistically significant. All statistical analyses were conducted using SPSS version 17.00 for Windows (Chicago, Illinois, USA).

## Results

The main characteristics of the samples are displayed in Table 1. Controls were younger compared to both MCI groups and SCD (*p* < 0.001 and *p* 0.01, respectively). Formal education was significantly higher in controls compared to SCD (*p* 0.001), naMCI, and aMCI (*p* < 0.001 for both). As expected, MMSE scored significantly lower in both MCI groups compared to controls and SCD (*p* < 0.001 for all comparisons).

Controls had the lowest prevalence of comorbidities compared to the other groups. With the only exception of CVD (no significant difference between controls and SCD), the difference in prevalence was highly significant (*p* < 0.001) for all these comparisons. The prevalence of female gender was not different between the groups.

**Fig. 1:**
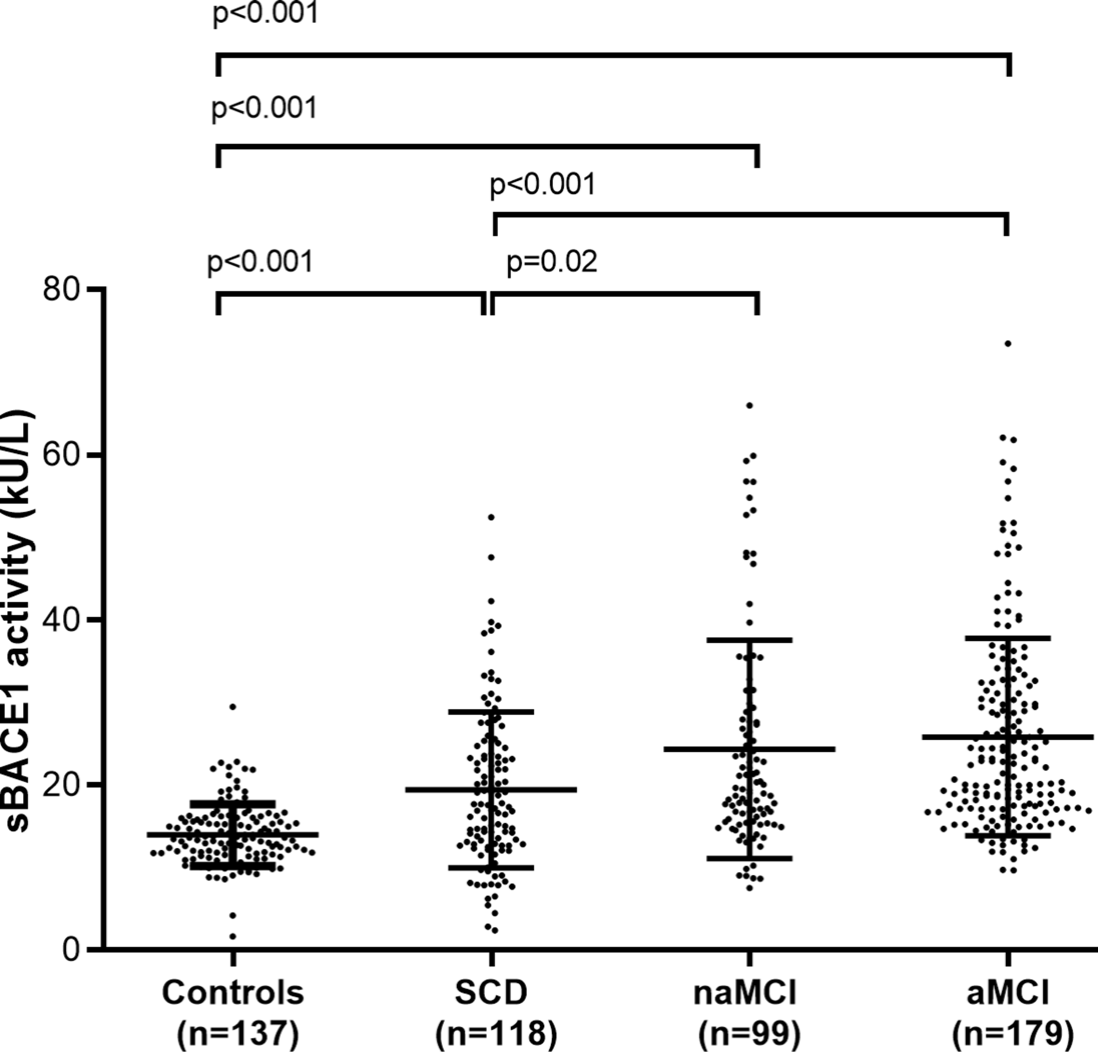
sBACE1 activity in controls and in subjects with subjective cognitive decline (SCD), non-amnestic mild cognitive impairment (naMCI), and amnestic mild cognitive impairment (aMCI). sBACE1 activity was significantly higher in SCD (+ 28%), naMCI (+ 42%), and aMCI (+ 45%) compared to controls. A significant increase in sBACE1 activity was observed in both MCI groups compared to SCD, although it was more evident in the amnestic group. On the contrary, no differences emerged between naMCI and aMCI. Overall, these results suggest that sBACE1 activity increases in function of subjective and objective cognitive impairment

As shown in Fig. 1, sBACE1 activity was significantly higher in SCD (+ 28%, *r* effect-size = 0.230), naMCI (+ 42%, *r* effect-size = 0.400), and aMCI (+ 45%, *r* effect-size = 0.406) compared to controls (*p* < 0.001 for all). A significant increase in sBACE1 activity was observed in both MCI groups compared to SCD, although it was more evident in the amnestic group (*p* 0.02 for SCD vs. naMCI, *r* effect-size = 0.208; and *p* < 0.001 for SCD vs. aMCI, *r* effect-size = 0.223). On the contrary, no differences emerged between naMCI and aMCI (*p* 0.43). Notably, all the pairwise differences detected at univariate analysis remained significant after adjustment for age, sex, MMSE, and comorbidities (overall ANCOVA, *p* < 0.0001). After adjustment for covariates the difference between controls and the other three groups was still highly significant (*p* < 0.001; controls vs. SCD, *r* effect-size = 0.251; controls vs. naMCI, *r* effect-size = 0.327; controls vs. aMCI, *r* effect-size = 0.392), whereas the significance slightly decreased for the comparisons SCD vs. naMCI (*p* = 0.03, *r* effect-size = 0.196), and SCD vs. aMCI (*p* = 0.01, *r* effect-size = 0.227).

**Fig. 2:**
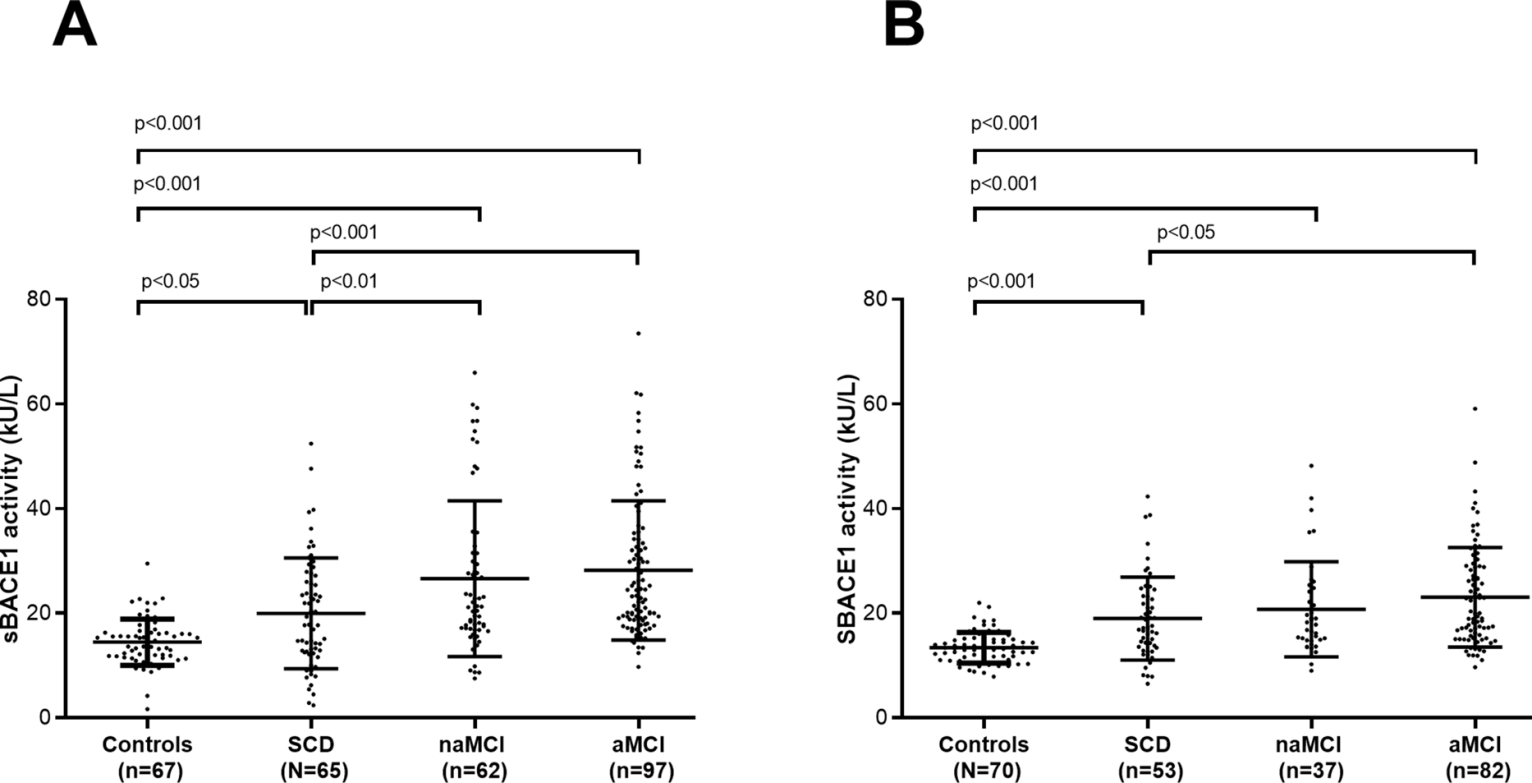
sBACE1 activity in controls and in subjects with subjective cognitive decline (SCD), non-amnestic mild cognitive impairment (naMCI), and amnestic mild cognitive impairment (aMCI) among women (**A**) and men (**B**). The levels of sBACE1 activity significantly increased in SCD, naMCI and aMCI compared to controls in both men and women even though they were more pronounced in the female subset, especially for the two MCI sub-groups. sBACE1 activity was increased also naMCI and aMCI, compared to SCD, but this change was more evident in women than in men. Thus, the change in sBACE1 activity seen in the whole sample appear to be mostly driven by women

Since sex was the only covariate retaining a significant association with sBACE1 activity in the ANCOVA model (*p* < 0.001, data not shown), we examined the trend of this marker in males and females, separately. The differences between controls and SCD, and naMCI and aMCI, were maintained in both men and women (*p* 0.02 for controls vs SCD in women, and *p* < 0.001 for all the others) even though they appeared to be more pronounced in women, especially for the two MCI sub-groups (Fig. 2). Similarly, the difference in sBACE1 activity between SCD, naMCI, and aMCI were more evident in women (*p* < 0.01 and *p* < 0.001, respectively) than in men, where the marker retained its significance only with respect to aMCI (*p* < 0.05). The discrepancies between men and women were mostly since sex mostly influenced sBACE1 activity in the MCI sub-groups; indeed, in women, we observed an increase in sBACE1 activity of 22% (*p* < 0.01) in aMCI and 19% in naMCI (*p* < 0.05) compared to the respective subgroups in men. On the contrary, the increase of sBACE1 activity in women compared to men was modest and not significant in controls and SCD (10% and 5%, respectively).

**Fig. 3:**
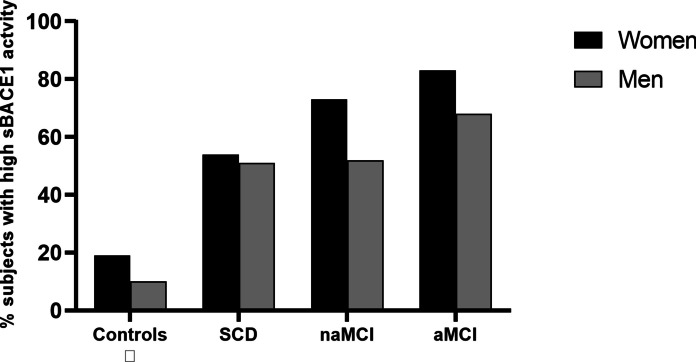
Percentage of men or women with “HIGH” sBACE activity (above 16.9 kU/L) in controls and in subjects with subjective cognitive decline (SCD), non-amnestic mild cognitive impairment (naMCI), and amnestic mild cognitive impairment (aMCI). The prevalence of subjects with elevated sBACE1 activity is higher in women in all four study groups, in particular in naMCI and aMCI

For each group of subjects, we also calculated the percentage of women or men with sBACE1 higher than the cutoff value of 16.9 kU/L [23]; this value was previously identified as able to discriminate between subjects with high and low likelihood of receiving a diagnosis of dementia. As displayed in Fig. 3, a trend toward an increase in the percentage of subjects with high sBACE1 activity from controls to SCD to naMCI to aMCI was noted, and this was particularly evident in women. We also found a clear difference between sex in naMCI and aMCI, with higher percentage of subjects with high sBACE1 activity in women compared to men (73% vs. 52% and 83% vs. 68%, respectively). Notably, the same trend emerged in controls (19% of women vs 10% of men), but not in subjects with SCD.

## Discussion

In this study, we extended the previous findings showing that sBACE1 activity could be an early biomarker for AD even at the stages of SCD and MCI. In particular, we and others have shown that sBACE1 activity is already elevated in MCI, especially in those progressing to AD over time [10, 18]. Here, we report for the first time that sBACE1 activity might be increased in older individuals affected by SCD even before the appearance of an objective cognitive impairment. In particular, the activity of sBACE1 was on average 25% higher in SCD than controls, and almost 50% of subjects with SCD demonstrated an elevated sBACE1 activity. Notably, the difference we found between controls and SCD individuals remained significant after adjusting for potential confounding factors including age, sex, MMSE, and comorbidities as resulted from the multivariate analysis (i.e., ANCOVA).

Over the last two decades, BACE1 has been attracting the interest of several clinical researchers due to its role in amyloid-beta (Aβ) homeostasis, and its potentials as pharmacological target and diagnostic/prediction biomarker of AD [24, 25]. Despite the recent burst of publications on the topic, the actual impact of BACE1 in Aβ formation and clearance is not well defined yet. BACE1 has traditionally been identified as the key enzyme in the amyloidogenic cascade, responsible for cleaving amyloid precursor protein (APP) at the β site, leading to the production of sAPPβ [25, 26]. This peptide is then cleaved into the neurotoxic and pro-aggregating Aβ40 and Aβ42 [26]. This pathogenic scenario justifies the investigation of BACE1 inhibitors as potential therapeutics for AD patients. However, all such trials have failed due to lack of efficacy and/or safety concerns [27]. One possible explanation for these failures has been recently suggested by our in vitro studies, which confirm the previously postulated dual role of BACE1 as both an amylogenic and an amyloidolytic player [28]. Indeed, it has been shown that classically employed inhibitors could reduce the levels of Aβ40 and Aβ42 but also decrease the levels of the amyloidolytic product Aβ34 [28], which is non-toxic, non-aggregating, and a potential in vivo indicator of amyloid clearance [8, 9]. Interestingly, in cells overexpressing BACE1, which mimic conditions seen in the AD brain, only the amyloidolytic activity was affected at relatively low inhibitor concentrations [28]. This aligns with previous studies showing that while levels of both BACE1 and Aβ34 are approximately doubled in AD brain tissue, deleting one BACE1 gene copy in mice reduces Aβ34 levels by half, proving a correlation between BACE1 protein levels and its amyloidolytic activity [8]. According to this body of evidence, the increase in BACE1 activity observed in the blood of SCD patients may be an early defense mechanism to decrease the levels of Aβ40 and Aβ42. It is tempting to speculate that stimulating BACE1’s amyloidolytic activity while halting Aβ production, without decreasing Aβ clearance, could be a promising therapeutic approach with new BACE1 modulators.

Individuals with SCD feature a self-experienced persistent decline in cognitive capacity, compared with a previously normal cognitive status, and normal performance on standardized cognitive tests used to classify MCI [1]. There is a large amount of published data suggesting that SCD might represent a prodromal marker of dementia, at least in some individuals. A meta-analysis has shown that 14% of older subjects with SCD progress to dementia and 27% to MCI [29]. These data have been recently updated by subsequent meta-analysis on 37 studies. The mean conversion rate of SCD was 7.3% and 4.9% to all-cause dementia and AD, respectively [30]. Although data differ among studies, these percentages remain much higher compared to those observed in the normal population of the same age [1].

Finding biomarkers able to identify the SCD subjects at greater risk of future MCI or dementia is a relevant research and clinical topic. In a recent study, SCD subjects with abnormal levels of Aβ biomarkers (according to the ATN system: CSF Aβ42, and/or amyloid PET) showed an increased risk of clinical progression to dementia and a steeper decline in many cognitive domains compared to participants with normal biomarkers [31]. Similarly, cerebral Aβ-protein and CSF total tau were found to be predictors of SCD progression to dementia in Li’s meta-analysis [30]. Of interest, BACE1 has already shown some potentials in this context. Indeed, Vergallo et al. focused attention only on SCD individuals, and found that high plasma BACE1 activity was associated with progressive neurodegeneration in brain regions mostly involved in AD pathology [19]. These findings may suggest that the SCD individuals of our cohort exhibiting increased levels of sBACE1 activity may have a higher risk of developing dementia; aMCi and naMCI patients had higher levels of sBACE1 activity compared to either controls or SCD. This stage-dependent increase strengthens the evidence of an inverse correlation of this marker with objectively assessed cognitive status (in agreement with previous studies, sBACE1 activity was weakly correlated with MMSE, *r* − 0.16, *p* < 0.001, data not shown). This stepwise increase is chiefly influenced by sex. Indeed, sBACE1 activity significantly increased in aMCI compared to SCD in both sexes, while it increased in naMCI compared to SCD only in women. This outcome reflects the fact that women with MCI have much higher levels of sBACE1 compared to men with MCI, whereas no differences between the two sexes were detected in SCD individuals.

The impact of sexual dimorphism in BACE1 levels has been already documented in animal and human (including post-mortem) studies and has been linked to the greater risk of dementia in women compared to men [32, 33]. The advanced hypothesis is that the menopause-related decline of estrogens, which are able to downregulate BACE1 gene transcription [34], could lead to a spark in BACE1 expression. Intriguingly, both animal and human studies showed an earlier and more severe AD pathology in females than in males [35–38]. Thus, it is tempting to speculate that the significantly higher levels of sBACE1 activity observed in women with MCI, compared to men with the same condition, could represent a brain defense reaction which has to cope with the initial increase of amyloid burden [39].

Finally, we should also acknowledge some important limitations of the study. First, the study was cross-sectional, thereby precluding our ability to establish any cause/effect relationship between BACE1 and SCD or MCI. Indeed, by definition, this type of design provides only a snapshot of a population at a specific point in time. Consequently, it limits our ability to assess the predictive accuracy of sBACE1 for future cognitive decline or to confirm its role in the early detection of the disease. A longitudinal approach is the only way to clarify this link and to evaluate whether sBACE1 could predict the progression of SCD to MCI and AD. Future studies should specifically focus on following SCD patients for up to 10 years (time period suggested by the results in [30]). This type of study, which involves monitoring participants over time, allows for the observation of event sequences. If we find that higher baseline levels or over time increases in sBACE1 in SCD individuals precede the onset of AD, it could suggest that alterations in this enzyme may contribute to disease development. Such an investigation could reveal the true clinical value of sBACE1 as a disease predictor and, ideally, as a marker for identifying individuals who might benefit from, yet to be developed, preventive drugs (like the aforementioned BACE1 modulators). Second, we cannot exclude that biases or not assessed variables might influence our findings or falsely demonstrate an apparent association. Indeed, there could be unassessed confounding factors such as anthropometric parameters (e.g., body mass index and waist circumference), diet, physical exercise, hormones, mediators of inflammation and vascular function, etc. that may distort or undermine the validity and generalizability of the results. Unfortunately, the potential biological modulators of sBACE1 are not well understood. Future studies involving the general population should aim to address this gap in knowledge. However, several potential confounding factors (e.g., age, gender, MMSE) were considered in our analyses and the observed increase in sBACE1 activity was apparently unaffected by these factors. Third, the individuals enrolled in this study were not characterized by neuroimaging and CSF biomarkers of AD; thus, misclassification of some of them cannot be ruled out. We are aware that the unavailability of these information affects the clinical relevance of our findings.

Nonetheless, we believe that the major strength of our study resides in the novel application of blood-based biomarkers like sBACE1 in subjects with SCD, a possible very early pre-clinical stage of dementia. In fact, the biomarkers employed for the clinical confirmation of probable AD like Aβ42, t-tau, and p-tau either require a CSF sample, since this is the only body fluid where they have been validated [40], or need expensive assays employing the Simoa™ or Elecsys® technology for their robust assay in blood [41]. Therefore, we consider our method for the detection of sBACE1 as an important positive feature of our research. It is an easy-to-perform assay that can be used in a standard biochemistry laboratory, and is much more cost-effective than the other assays for Aβ42, t-tau, and p-tau isoforms employing the Simoa™ or Elecsys® technology. Moreover, as previously documented, the assay has excellent analytical performance (low intra- and inter-assay variability) and showed high diagnostic accuracy for AD and MCI [16]. Indeed, we observed that sBACE1 can effectively distinguish between cognitively healthy individuals and patients with MCI who are progressing to AD, as well as those already diagnosed with AD (area under curve, AUC > 0.9700 for both groups) [16]. Notably, sBACE1 outperformed the widely used plasma Aβ 1–42 and plasma Aβ 40/42 ratio [16]. However, we recognize that future studies should directly compare the predictive performance of sBACE1 in SCD subjects with other well-established blood-based AD biomarkers.

Once sBACE1 is definitively validated as both a diagnostic and predictive biomarker in population ranging from SCD to MCI, one of its primary uses could be in pre-screening individuals for recruitment in clinical trials. Indeed, a substantial body of evidence suggests that incorporating blood-based biomarkers for AD at various stages of trial enrolment could reduce the need for lumbar punctures, and MRI and PET scans, leading to significant time and cost savings. The cost-effectiveness of sBACE1 could make it suitable for screening the general population to detect individuals at risk of AD.

Most of the treatment options currently being explored for this disease are effective when started early, ideally before clinical symptoms appear. Therefore, population screening could facilitate early intervention and potentially even prevention strategies.

In conclusion, our results provide evidence in support of a potential use of sBACE1 activity as a new biomarker for blood-based screening of cognitively healthy individuals at clinical risk for MCI and dementia. Further research is needed to ascertain the real impact of BACE1 in Aβ homeostasis and the reliability of sBACE1 as predictor of dementia in SCD individuals.

## Supplementary Information

Below is the link to the electronic supplementary material.

Supplementary file1 (TIF 277 KB)

## Data Availability

The Excel database utilized in this study is available upon request for readers, reviewers, and editors.
