# Supplementary figures and images for: Serum β-secretase 1 (sBACE1) activity in subjective cognitive decline: an exploratory study
Source: GeroScience. 2025 Jan 20;47(3):4109–20. doi: 10.1007/s11357-025-01523-x (PMC12181534; doi:10.1007/s11357-025-01523-x)

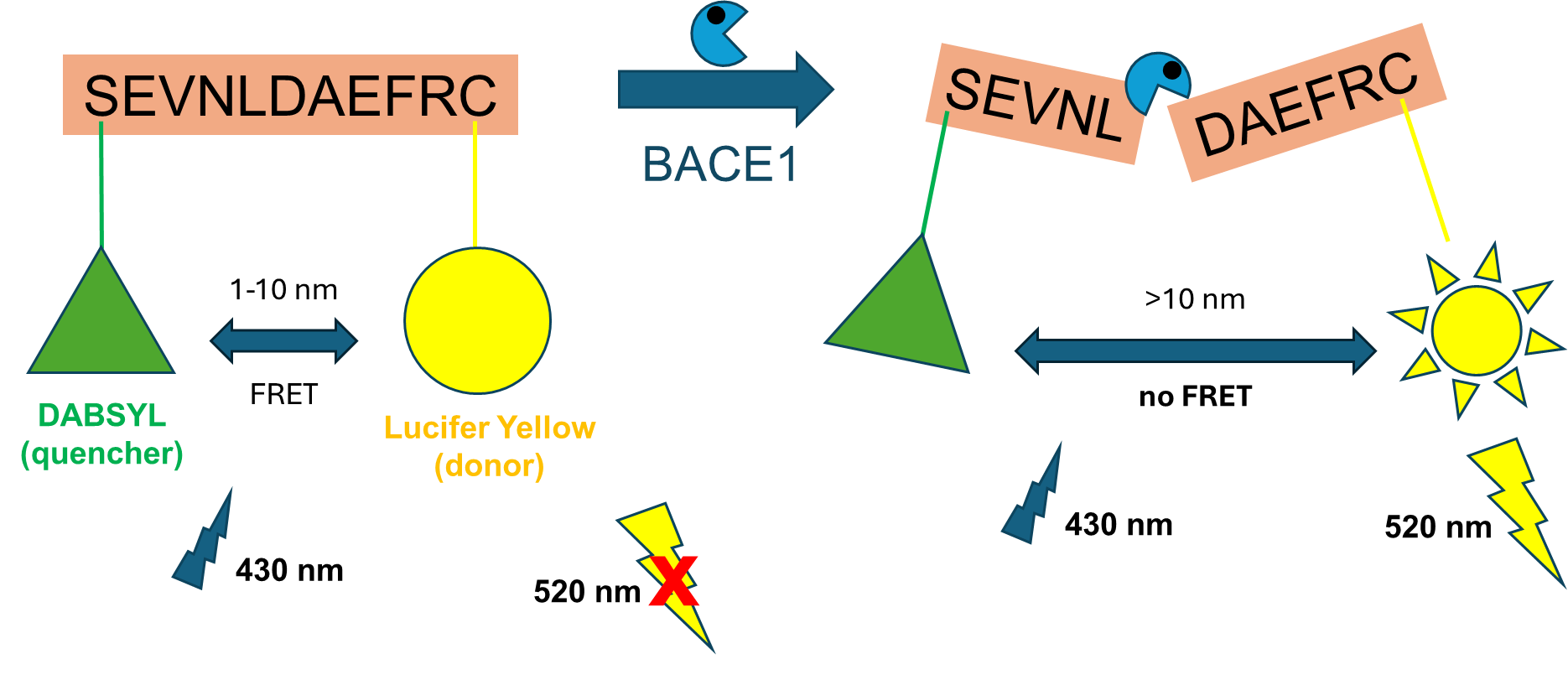

Supplement: Supplementary file 1 — Supplementary file1 (TIF 277 KB) [file 11357_2025_1523_MOESM1_ESM.tif]
